# Supplementary material for: A validated protocol to UV-inactivate SARS-CoV-2 and herpesvirus-infected cells
Source: PLoS One. 2023 May 10;18(5):e0274065. doi: 10.1371/journal.pone.0274065 (PMC10171616; doi:10.1371/journal.pone.0274065)
Supplement: S1 File — Protocol describing UV inactivation of infected cells. Also available at https://doi.org/10.17504/protocols.io.81wgb676qlpk/v1. (PDF) [file pone.0274065.s001.pdf]

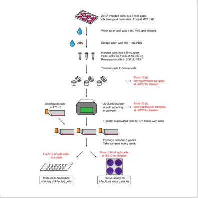

A validated protocol to UV-inactivate SARS-CoV-2 and herpesvirus-infected cells

Timothy K. Soh[1,2,3,4], Susanne Pfefferle[5,6], Stephanie Wurr[5,7], Ronald von Possel[5,8], Lisa Oestereich[5,7], Toni Rieger[5], Maria Rosenthal[1,5,9], Jens B. Bosse[1,2,3,4]

[1]Centre for Structural Systems Biology, Hamburg, Germany;

[2]Hannover Medical School, Institute of Virology, Hannover, Germany;

[3]Cluster of Excellence RESIST (EXC 2155), Hannover Medical School, Hannover, Germany;

[4]Leibniz Institute of Virology (LIV), Hamburg, Germany;

[5]Department of Virology, Bernhard-Nocht Institute for Tropical Medicine, Hamburg, Germany;

[6]University Center Hamburg-Eppendorf (UKE), Institute for Medical Microbiology, Virology and Hygiene, Hamburg, Germany;

[7]DZIF German Center for Infection research, Partner site Hamburg-Lübeck-Borstel-Riems;

[8]Department of Tropical Medicine and Infectious Diseases, Center for Internal Medicine, University of Rostock, Rostock, Germany;

[9]Fraunhofer Institute for Translational Medicine and Pharmacology (ITMP), Discovery Research ScreeningPort, Hamburg, Germany

Maria Rosenthal: rosenthal@bnitm.de;

Jens B. Bosse: jens.bosse@cssb-hamburg.de;

1 Works for me

Reserved DOI:
10.17504/protocols.io.81wgb676qlpk/v1

Jens B Bosse

ABSTRACT

Downstream analysis of virus-infected cell samples, such as reverse transcription polymerase chain reaction (RT PCR) or mass spectrometry, often needs to be performed at lower biosafety levels than their actual cultivation, and thus the samples require inactivation before they can be transferred. Common inactivation methods involve chemical crosslinking with formaldehyde or denaturing samples with strong detergents, such as sodium dodecyl sulfate. However, these protocols destroy the protein quaternary structure and prevent the analysis of protein complexes, albeit through different chemical mechanisms. This often leads to studies being performed in over-expression or surrogate model systems. To address this problem, we generated a protocol that achieves the inactivation of infected cells through ultraviolet (UV) irradiation. UV irradiation damages viral genomes and crosslinks nucleic acids to proteins but leaves the overall structure of protein complexes mostly intact. Protein analysis can then be performed from intact cells without biosafety containment. While UV treatment protocols have been established to inactivate diluted viral solutions, a protocol was missing to inactivate crude infected cell lysates, which heavily absorb light. In this work, we develop and validate a UV inactivation protocol for SARS-CoV-2, HSV-1, and HCMV-infected cells. A fluence of 10,000 mJ/cm^2 with intermittent mixing was sufficient to completely inactivate infected cells, as demonstrated by the absence of viral replication even after three sequential passages of cells inoculated with the treated material. The herein described protocol should serve as a reference for inactivating cells with these or similar viruses and allow for the analysis of protein quaternary structure from *bona fide* infected cells.

PROTOCOL INFO

Timothy K. Soh, Susanne Pfefferle, Stephanie Wurr, Ronald von Possel, Lisa Oestereich, Toni Rieger, Maria Rosenthal, Jens B. Bosse . A validated protocol to UV-inactivate SARS-CoV-2 and herpesvirus-infected cells. **protocols.io** https://protocols.io/view/a-validated-protocol-to-uv-inactivate-sars-cov-2-a-b32mqqc6

FUNDERS ACKNOWLEDGEMENT

Deutsche Forschungsgemeinschaft
Grant ID: EXC 2155 – project number 390874280

Deutsche Forschungsgemeinschaft
Grant ID: GRK 2771 project number 453548970

Wellcome Trust
Grant ID: Collaborative Award (209250/Z/17/Z)

Bundesministerium für Bildung und Forschung
Grant ID: grant 01KI2019

KEYWORDS

SARS-CoV-2, herpes, HSV-1, HCMV, UV crosslinking, UV inactivation, inactivation validation

CREATED

Jan 19, 2022

LAST MODIFIED

Aug 15, 2022

PROTOCOL INTEGER ID

57133

GUIDELINES

This protocol was validated for $2x10^6$ Vero E6 cells infected with SARS-CoV-2 at MOI 0.01, $5x10^5$ Vero B4 cells infected with HSV-1 at MOI 3, and $2x10^5$ HFF-1 cells infected with HCMV at MOI 3 in an individual 6-well.

MATERIALS TEXT

phosphate-buffered saline (PBS)
Incidin Plus

infected cells
cell scraper
microcentrifuge tube
CryoELITE Tissue Vial (Wheaton, #W985100)

P1000 micropipette
microcentrifuge
UVP Crosslinker (CL-3000, Analytik Jena)

SAFETY WARNINGS

Handle infectious materials within the appropriate containment facilities. Use UVP Crosslinkers in accordance with the manufacturer's guidelines.

BEFORE STARTING

Begin with infected cells in a 6-well plate.

UV inactivate infected cells

1 Wash each well with 1 mL PBS — 1m

2 Scrape each well ($2x10^6$ cells) into 1 mL PBS — 1m

3 Transfer cells into a 1.5 mL tube — 1m

4 Pellet cells at **16000 x g, 4°C, 00:01:00** 1m

5 Resuspend cells in 200 µL PBS 1m

6 Transfer cells to a tissue vial 1m

7 254 nm UV irradiation of vials 24m

7.1 Irradiate vials with 2,500 mJ/cm^2 5m

7.2 Mix the cell solution with a micropipette 1m

7.3 Repeat irradiation 3 additional times for a total of 10,000 mJ/cm^2 18m

8 Screw the lids on the vials 1m

9 Disinfect the outside of the tissue vials by wiping with Incidin Plus 1m
