# Supplementary material for: A validated protocol to UV-inactivate SARS-CoV-2 and herpesvirus-infected cells
Source: PLoS One. 2023 May 10;18(5):e0274065. doi: 10.1371/journal.pone.0274065 (PMC10171616; doi:10.1371/journal.pone.0274065)
Supplement: Attachment — Submitted filename: Response to Reviewers.pdf [file pone.0274065.s002.pdf]

Response to Reviewers

We sincerely thank all reviewers for their critical reading of our manuscript and their insightful and fair suggestions. Below is our response to the reviewers' comments (highlighted in light blue). References have been added to the reference list. Line numbers refer to the document with tracked changes.

Reviewer #1: In the submitted manuscript, authors seek to validate a UV inactivation protocol for virally infected cells. UV exposure of virus suspensions is very effective for the stabilization of protein conformations and activity while rendering highly pathogenic viruses incapable of propagating infection. There are many limitations to UV treatment, with particular regards sample complexity. Evaluation of UV conditions to inactivate virus within cells has not been previously performed. Authors compare the effectiveness of UV exposure for 3 viruses; HSV-1, CMV, and SARS-CoV-2. Using plaque assay and immunofluorescence assays, they demonstrate that 10,000 mJ/cm^2 of UV 257nm irradiation will completely eliminate infectivity from infected cell suspensions of the 3 viruses.

Overall, the study is framed well with the introduction and discussion. The evaluation of residual infectivity with multi-week passage of exposed cells is rigorous. Testing of viral infectivity by both plaque/FFA assay and immunofluorescence detection of infected cells is equally robust. Experimentally, there is a gap regarding the "sufficiency" of 10,000 mJ exposure and the equivalency of inactivation from the 3 viruses. While inactivation conditions are validated for HSV-1, CMV, and SARS-2, it is unclear if this much exposure is necessary or possibly detrimental to downstream processes.

In addition, the discussion needs to be enhanced with a brief comparison of UV inactivation results between RNA and DNA viruses. The results section needs additional information and explanation of experimental rationale and interpretation. There are additional minor details that need to be addressed.

We revised the manuscript according to the reviewer's suggestions. We explained our rationale for establishing inactivation conditions using HSV-1 as a baseline (line 174). We also included a discussion of UV inactivation of DNA versus RNA viruses (line 264). The rationale for mixing during irradiation, the choice of tissue vial, and further experimental details have been added to the results section (line 160, 177, 178).

Major Concerns:

1) Equivalency of DNA and RNA viruses to UV inactivation. The sensitivity of viruses to UV irradiation is variably reported in the literature. Authors are correct in identify differences in containers, distance, etc. But equally, DNA and RNA viruses will have different reactions to UV irradiation exposure. DNA viruses have a limited capacity to repair lesions induced by UV crosslinking that RNA viruses don't necessarily have. UV sensitivity is only tested for HSV-1 in figure 1, while all subsequent experiments are performed at the maximal UV dose. Authors should briefly address the possible discrepancy between nucleic acid types in the discussion. Better would be a comparison of sensitivity to UV inactivation for SARS-CoV-2 infected cells.

We added a section to the discussion where we summarize the literature on UV sensitivity of DNA versus RNA as well as the potential influence of DNA repair mechanisms (line 264).

2) Line 168 "We found that the UV dose needed to inactivate infected cells was much higher than…" This line is difficult to reconcile. First, evaluation of UV sensitivity of cell-free virus suspensions would be a necessary comparison in your system to declare that infected cells require more. Secondly, for CMV and SARS-2 intermediate UV exposures are not tested to demonstrate that 10,000 mJ is necessary. Emphasizing that you have validated conditions of complete inactivation is all that is proven. Further comparison of relative dosing is not appropriate in the results or discussion.

We thank the reviewer for pointing this out. We changed the language to better reflect that we used a sufficient dosage that is safe for all tested viruses but which might not necessarily be the lowest required dosage for inactivation for individual viruses (line 182, 191, 229).

3) Explanation of results – The results section is lacking in explanation of experimental rationale. Multiple questions can/should be addressed in this section.

a. What is the geometry of the cell suspension in the vial. Vial dimensions are very large (5 mL max volume) while volume is only 200 uL. Did authors attempt other volumes of resuspension? As they note, cell density will influence the optical density which could reduce UV efficacy through absorbance and shading. Was the drop spread out? What was the depth? Did a larger volume change UV inactivation efficiency?

We added this information to the results section (line 162). Briefly, the sample was spread out on the bottom of the vial, but given the small volume, it still formed a droplet. This droplet has a maximal depth of 5 mm. During optimization, cells were resuspended in 100 µL to 300 µL prior to UV treatment. There was no clear difference in efficiency. As about 20-40 µL of sample volume is lost during handling due to adherence to the container walls, 200 µL was chosen as a compromise to minimize the volume while reducing the unrecovered fraction.

b. Why was a multi-week passaging of potentially infected cells carried out? Was plaque assay/IFA performed from UV inactivated cell suspensions directly? The former is very rigorous but seems somewhat unnecessary.

The multi-week passaging is required by our institute's biosafety regulations. We noted this in the results section (line 199). We appreciate the recognition of the rigour of this requirement. A plaque assay/IFA was not performed directly on the UV-treated material since it would be measured after the material had an opportunity to replicate.

Minor Concerns:

1) Line 29 – "However, these protocols destroy…" It is unclear why equivalency between formaldehyde cross-linking and sds denaturation are being made here.

The reviewer is correct. These chemicals function through different mechanisms. We modified the manuscript accordingly to emphasize that both compounds obstruct the identification of physiological protein complex compositions, although through different mechanisms (line 33). High concentrations of formaldehyde that are sufficient to inactivate the sample will crosslink proteins that are not part of the same complex, and reversal of the crosslinker through heat will destroy the protein complex interactions.In contrast, SDS will directly disrupt the protein interactions.

2) Line 170 – "This was likely due to cells…" The geometry of the container also needs to be taken into consideration. The top of cell suspension is the area receiving UV irradiation and will rapidly lose efficacy within the solution. What was the depth of solution in the vial?

In accordance with the reviewer's earlier comment concerning how our dose represents a sufficient but not minimal dose for SARS-CoV-2, this paragraph was changed to compare HSV-1 to published values (line 231).

3) Line 187 "certain downstream assays." It is unclear at this point in the manuscript what these downstream assays would entail. Subsequent explanation of CLIP and other nucleic acid association assays are reasonable, but disconnected from this statement.

An example is now given to provide context (line 255).

4) Figure 2 title – "Pre-study experiment" is not informative and should be revised. The higher LOD is not sufficiently explained but presumptively reflects the 10 uL sample volume being dilute 1:100 for initial analysis? It is unclear if this "n" refers to the number of replicate experiments or samples evaluated at each UV inactivation dose.

We changed the title to "Mixing cells during irradiation increases inactivation efficiency" (line 184). N is now defined as "Each dose curve was performed with n=1" (line 189). An explanation of the different LoD was added to the methods section (line 94).

5) Supplemental Instructions line 23 – The last step should indicate the agent of disinfection and time, i.e. 70% ethanol for 5 minutes.

We changed the text to “Disinfect the outside of the tissue vials by wiping with Incidin Plus.“

Reviewer #2: Nice work. Please spell out short from in first line of Abstract. UV light can damage proteins. This may be something to keep in mind in future work. The UV dose you are using is quite high. It would be good if you could reduce it.

We have defined reverse transcription polymerase chain reaction (RT PCR) (line 27) and added an additional section where we discuss the used UV dose (line 316).
